# Uncommon Presentation of Cystic Fibrosis: A Case Report and Literature Review

**DOI:** 10.7759/cureus.45186

**Published:** 2023-09-13

**Authors:** Majed Abu Sirhan, Michael Kalinin, Lior Cohen, Evgenia Gurevich

**Affiliations:** 1 Pediatric Department, Barzilai University Medical Center, Ashqelon, ISR; 2 Pediatric Intensive Care Unit, Barzilai University Medical Center, Ashqelon, ISR; 3 Genetics Institute, Barzilai University Medical Center, Ashqelon, ISR; 4 Faculty of Health Sciences, Ben-Gurion University of the Negev, Beersheva, ISR

**Keywords:** hyponatremia, • dehydration, genetic screening, hypokalemic hypochloremic metabolic alkalosis, cystic fibrosis (cf)

## Abstract

Cystic fibrosis (CF) is a multiorgan disease, caused by autosomal recessive (AR) mutations in the cystic fibrosis transmembrane regulator (CFTR) acting primarily as a chloride channel. CF is most commonly diagnosed in Caucasian populations. Common clinical presentations in pediatric patients include chronic cough, respiratory tract infections such as pneumonia, digestive symptoms, and stunted growth, and malnutrition due to gastrointestinal malabsorption and pancreatic insufficiency. Excessive sweat sodium chloride losses due to dysfunctional sweat glands in CFTR result in volume contraction and secondary hyperaldosteronism leading to renal potassium losses and metabolic alkalosis. Hypokalemic hypochloremic metabolic alkalosis is a known but uncommon presenting sign of the disease, documented as pseudo Bartter syndrome. Common mutations in the *CFTR* gene are now included in prenatal genetic screening programs. We describe the case of an infant of African descent with normal prenatal screening who presented with severe hypokalemic hypochloremic metabolic alkalosis and was diagnosed with CF with further genetic confirmation of the diagnosis.

## Introduction

Cystic fibrosis (CF) is a multiorgan disease, caused by autosomal recessive (AR) mutations in the *CFTR* gene, which regulates the movement of chloride ions across cell membranes [1]. CF is most commonly diagnosed in Caucasian populations but can affect other ethnic groups as well, with an incidence of one in 4100 live births in the United States, one in 2500-3500 live births in individuals of European descent, and an average of one in 5000 in Israeli population depending on ethnic subgroups [2,3]. Common clinical presentations in pediatric patients include chronic cough, respiratory tract infections such as pneumonia, and digestive symptoms such as stunted growth and malnutrition due to gastrointestinal malabsorption and pancreatic insufficiency. Hypochloremic hypokalemic metabolic alkalosis is a known but uncommon presenting sign of the disease, documented as pseudo Barter syndrome [4]. It is caused by excessive sweat sodium chloride losses due to dysfunctional CFTR protein, leading to volume contraction, secondary hyperaldosteronism, and the development of hypokalemia and hypochloremic metabolic alkalosis. CF is now included in neonatal screening programs globally, allowing early detection, management, and improved clinical outcomes [5].

In this report, we describe a case of an infant of African descent with normal prenatal screening who presented with severe hypokalemic hypochloremic metabolic alkalosis and was diagnosed with CF with further genetic confirmation of the diagnosis.

## Case presentation

A previously healthy five-month-old female presented in the pediatric emergency room with weight loss and dehydration without apparent cause, as there was no history of fever, vomiting, diarrhea, or poor feeding. She had been born in our hospital at term after normal pregnancy to healthy non-consanguineous parents of African descent. Prenatal carrier screening, which, in our country, includes common pathogenic variants in the *CFTR* gene (delF508, W1282X, N1303K, G542X,3849+10Kb, 1717+1G>A,3121-1G>A, Y1092X, I1234V, W1089X, S549R, G85E, 405+1G.A), was normal. She had no history of respiratory or gastrointestinal complaints, and her growth and development were normal. On admission, her physical examination was unremarkable except for moderate to severe dehydration signs such as dry mucous membranes, sunken eyes, decreased skin turgor, and decreased urine output. Her heart rate was 145/minute, blood pressure was normal (85/62 mmHg), her weight was 5.2 kg (third percentile), and after intensive rehydration with isotonic saline raised to 5.7 kg (10th percentile). Blood test results on admission revealed severe metabolic alkalosis (pH 7.68, partial pressure of carbon dioxide (PCO2) 30 mmHg, bicarbonate (HCO3) 35 mmol/L), hypochloremia (chloride (Cl) 73 mmol/L), severe hyponatremia (sodium (Na) 124 mmol/L), and hypokalemia (K 2.6 mmol/L). Using an algorithm for differential diagnosis of normotensive hypokalemic hypochloremic metabolic alkalosis, we ruled out urinary and stool chloride losses (urine chloride <15 mmol/L, stool chloride 12 mmol\L). A sweat chloride test was positive (125 mmol/L, Na<40 mmol/L), suggesting the diagnosis of CF. Laboratory data for our patient are presented in Table 1. 

**Table 1:** Laboratory findings in our patient Na: sodium; K: potassium; Cl: chlorine; PCO2: partial pressure of carbon dioxide; HCO3: bicarbonate

	Result	Reference range
Na blood (mmol/L)	124	136-145
K blood (mmol/L)	2.6	3.5–5.10
CI blood (mmol/L)	73	98-107
pH	7.68	7.32–7.43
PCO2 (mmHg)	30	38-48
HCO3 (mmol/L)	35	22-29
CI urine (mmol/L)	<15	n/a
CI Stool (mmol/L)	12	n/a
Sweat CI (mmol/L)	125	<29

The results of the genetic panel for common *CFTR* mutations were negative. *CFTR *next-generation sequencing (NGS) revealed a homozygous missense variant in exon 4, specifically c.416A>T; p.His139Leu; chr7:117171095A>T, indicated as deleterious by several predictive bioinformatic tools.

**Figure 1:**
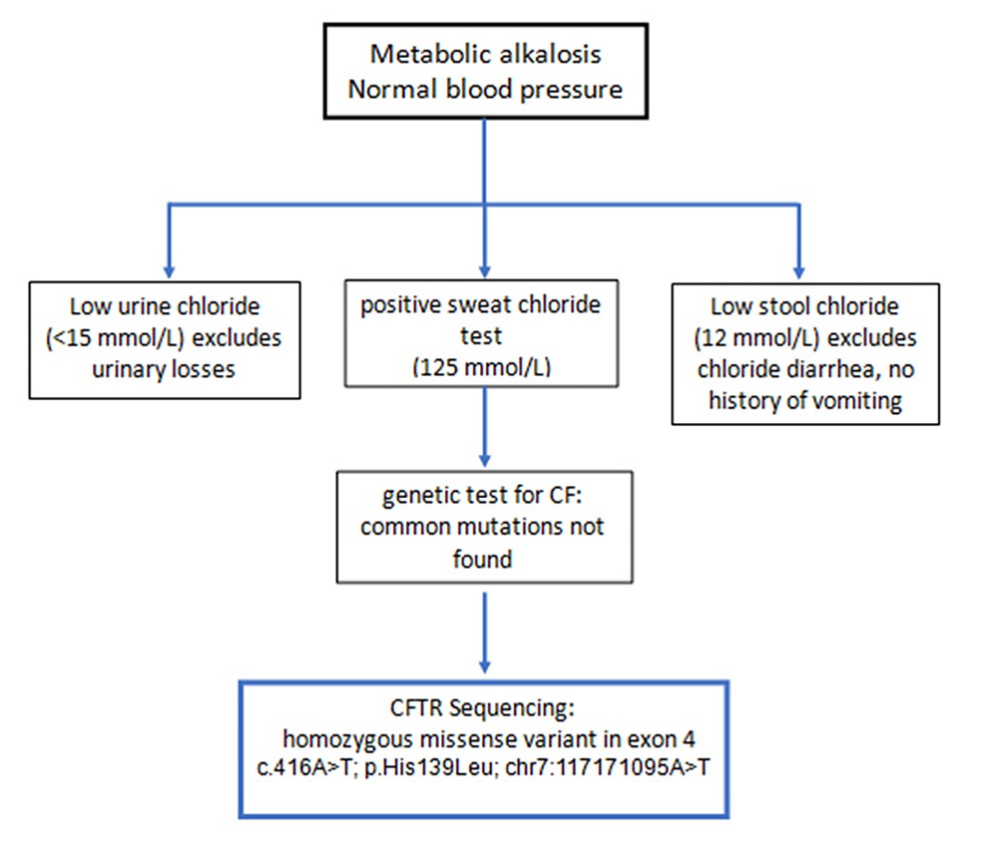
Diagnostic algorithm of normotensive metabolic alkalosis in our patient in the pediatric ward

The diagnostic algorithm for our patient is presented in Figure 1.

During the hospital stay for five days, the patients' electrolyte abnormalities and dehydration were treated with isotonic saline and potassium supplementation. After normalization of electrolytes and blood gases and volume repletion, potassium supplementation was stopped, and the patient was discharged home with NaCl supplementation alone. Due to the patient's abnormal presentation, her four-year-old sister was brought to medical attention as she had a history of recurrent lung infections and failure to thrive. She underwent genetic testing for CF and the same mutation as in her sister was found. Both the patient and her sister were referred to the CF clinic in the tertiary medical center for further treatment and follow-up.

## Discussion

In this report, we describe the uncommon presentation of CF with hypokalemic hypochloremic metabolic alkalosis and the algorithm of the diagnosis in pediatric wards in an infant of African descent with normal prenatal screening results.

The approach to the differential diagnosis of metabolic alkalosis includes, as a first step, blood pressure measuring, which was normal in our patient. The differential diagnosis of normotensive hypokalemic hypochloremic metabolic alkalosis includes urinary chloride losses (Barter and Gitelman syndromes, diuretic use), gastrointestinal losses (vomiting, chloride diarrhea), and skin losses (CF). The use of this diagnostic algorithm in our case helped to make an appropriate diagnosis based on an abnormal sweat test after the exclusion of urinary and gastrointestinal chloride losses.

Metabolic alkalosis is an uncommon presentation of CF with only a few cases and case series previously described in the literature.

Beckerman et al. described a case series of 11 infants with CF, five of them were diagnosed between one and 12 months of age initially based on hypokalemia, hypochloremia, and metabolic alkalosis without major pulmonary and/or gastrointestinal symptoms [4]. In a case series of 103 children diagnosed with CF before the age of 12 months, 17 presented with hypokalemic hypochloremic metabolic alkalosis without prominent respiratory or gastrointestinal symptoms [6]. According to the study, predisposing factors for the development of metabolic alkalosis with hypoelectrolytemia in CF patients included early infant age, breastfeeding, delayed diagnosis, heat exhaustion, and the presence of *CFTR* mutations associated with a severe phenotype. Molecular screening for mutations in the *CFTR* gene in eight Sardinian children who presented with hypotonic dehydration and electrolyte imbalances, without pulmonary or pancreatic symptoms associated with CF, revealed the T3381 pathogenic variant in the *CFTR* gene, either in homozygosity or compound heterozygosity with another CF mutation. These findings highlight the unique relationship between this specific pathogenic variant and an uncommon presentation of CF with hypokalemic hypochloremic metabolic alkalosis [7]. In our patient, this unique presentation may be related to the extremely hot weather in the summertime in our country or possibly can be attributed to the specific mutation as described in the above case series.

CF is most commonly diagnosed in Caucasian populations. However, very little is known about CF in populations of African origin among whom it has been believed to be extremely rare [8]. Our patient was born to parents of African descent (Eritrean Ethiopian). Although consanguinity is uncommon in this population, which makes the possibility of the recessive homozygous disease extremely low, NGS analysis revealed a homozygous mutation in our patient. The identified variant has not been previously reported in the genome Aggregation Database (gnomAD) database (Broad Institute, Cambridge, Massachusetts). Several predictive bioinformatic tools have consistently indicated that this variant is likely to be deleterious. In ClinVar (National Center for Biotechnology Information, Bethesda, Maryland, United States), this variant has been reported three times (VCV53910) [9]. This mutation is also documented in the CFTR Mutation Database of the Hospital for Sick Children [10], as well as in the Ethiopian population in The Israeli Medical Genetic Database [11]. Furthermore, this mutation has been predominantly detected in families originating from the Gulf area, such as native Saudis and Bahraini regions [12]. It appears to be a founder mutation in Western Asia and Eastern Africa. According to the Centre for Arab Genomic Studies, it ranks among the 10 most common *CFTR* variants in the Gulf area, with a frequency of 6-7% [1,12]. Moreover, it seems to be a founder mutation in Africa as well. Individuals carrying this mutation have exhibited significant pulmonary disease in infancy, failure to thrive, and pancreatic insufficiency. Consequently, in the case of the patient in the current study, validation through Sanger sequencing has been requested for both the affected individual and her sister.

Prenatal screening program now includes common mutations in the *CFTR* gene, but they may miss rare mutations [13]. Therefore, a normal genetic screening result does not definitively rule out the possibility of CF. Nonetheless, prenatal screening remains a valuable tool for early and accurate diagnosis. This case highlights the fact that prenatal screening is not diagnostic and underscores the importance of maintaining a high level of clinical suspicion for CF in infants who exhibit suspicious symptoms, regardless of their prenatal screening results or ethnicity.

## Conclusions

This case report summarizes the diagnostic course in the pediatric ward to the final diagnosis of CF in an infant of African descent with uncommon presentation. Hypokalemic hypochloremic metabolic alkalosis may be the only presenting sign of CF. This diagnosis should be considered regardless of ethnicity and normal prenatal genetic testing.
